# Uterine mass after caesarean section: a report of two cases

**DOI:** 10.1186/s12884-020-03213-2

**Published:** 2020-09-03

**Authors:** Lin-yu Zhou, Xiao-dan Zhu, Jian Jiang, Tian-an Jiang

**Affiliations:** Department of Ultrasonography, The First Affiliated Hospital, College of Medicine, Zhejiang University, No. 79 Qingchun Road, Zhejiang Province 310003 Hangzhou, P.R. China

**Keywords:** Gestational trophoblastic neoplasia, Scar pregnancy, Caesarean section, Contrast-enhanced Ultrasound

## Abstract

**Background:**

Caesarean scar pregnancy (CSP) is a rare complication of caesarean delivery and a special type of ectopic pregnancy. Gestational trophoblastic neoplasia (GTN) is an uncommon complication of pregnancy. Early diagnosis of the two diseases is crucial because a delay or misdiagnosis can lead to increased maternal morbidity and mortality.

**Case presentation:**

We report two cases of uterine isthmus lesions with a previous caesarean section (CS). Two patients were misdiagnosed based on the first ultrasound exam. The first case of trophoblastic tumour was initially diagnosed as CSP, while the second case, which had a scar pregnancy, was misdiagnosed as GTN. The misdiagnoses were due to the particularity of the locations of the lesions in the two patients, complicating the ultrasound-based diagnosis and hindering early clinical diagnosis and treatment.

**Conclusions:**

A medical history, β-hCG measurements and transvaginal ultrasound are necessary to diagnose lesions in the lower anterior wall of the uterus early. However, when the location cannot be determined, magnetic resonance imaging (MRI) can be further performed to determine whether the lesion is located at the uterine scar. Combined with the degree of increased β-hCG, differentiate CSP, myometrial GTN or caesarean scar GTN is helpful.

## Background

The World Health Organization (WHO) reported that the mean caesarean section (CS) rate of 9 countries in Asia was 27.3% in 2010 and reached 46.2% in China [1]. The prevalence of CS differs between countries. The incidence of caesarean scar pregnancy (CSP) in China is higher than that in other countries. With the change in the one-child policy, more women have chosen to become pregnant again and will therefore face the risk of CSP [2]. CSP is a rare complication of caesarean delivery and a special type of ectopic pregnancy. CSP is refers to a pregnancy in which the embryo has implanted in the hysterotomy scar, is partly or completely located outside the uterine cavity, and is surrounded by myometrial and fibrous scar tissue [3]. The mechanism is speculated to involve the implantation of blastocysts in the tiny dehiscence tracts of the scar after prior caesarean section [4, 5]. Due to the fibrous nature of scar tissue, as the CSP enlarges, these inherently insufficient implant sites are at risk of rupture, placenta accreta spectrum, and haemorrhage. Ultrasound is the main imaging modality for the diagnosis of CSP, although accurate and early diagnosis of CSP may be difficult [6, 7]. In previous studies, approximately 15% of cases were originally misdiagnosed as cervical ectopic pregnancies, incomplete abortion, or low implantation of an intrauterine pregnancy [8, 9].

Failure to promptly diagnose CSP, and curettage treatment used can lead to uncontrollable haemorrhage and uterine rupture, which are life-threatening complications and may require hysterectomy to save the lives life of the mother [9, 10].

However, gestational trophoblastic disease can also occur in the lower anterior wall of the uterus, such as uterine scars or the cervix, thus complicating early diagnosis [11, 12]. Gestational trophoblastic neoplasia (GTN) is an uncommon complication of pregnancy. Considerable variation exists in the worldwide incidence of GTN, with the highest frequencies reported in Asia and the Middle East [13]. Approximately 50% of GTNs follows molar pregnancy, and the diagnosis is relatively easy. Postmolar GTN is typically diagnosed using β-hCG surveillance and the FIGO staging criteria [14]. However, the diagnosis of nonmolar GTN occuring after a spontaneous abortion, an ectopic pregnancy, or a term pregnancy is relatively difficult. Similar to CSP, the clinical presentation mainly includes abnormal vaginal bleeding, abortion, ectopic pregnancy and other gynaecological diseases, and the condition is associated with a lack of specificity in clinical practice and is also easily misdiagnosed. Research on trophoblastic tumours at the scar site has identified a set of diagnostic criteria for early diagnosis to avoid a missed diagnosis [15].

The two patients described here both have a history of scar pregnancy, and their clinical presentations were irregular vaginal bleeding. Larger lesions could be detected in the lower part of the anterior wall of the uterus by ultrasound. Unlike previous case reports, these two cases have similar ultrasound images showing a huge mass in the lower anterior wall of the uterus but completely different clinical diagnoses. Through comparison, we summarized our diagnosis and differential diagnosis considerations based on the similar images. When transvaginal ultrasonography (TVS) cannot confirm the specific location of the lesion and the diagnosis, magnetic resonance imaging (MRI) can be considered to further clarify the relationship between the mass and the uterine scar, which helps to achieve definitive diagnosis.

## Case presentation

### Case one

**Fig. 1:**
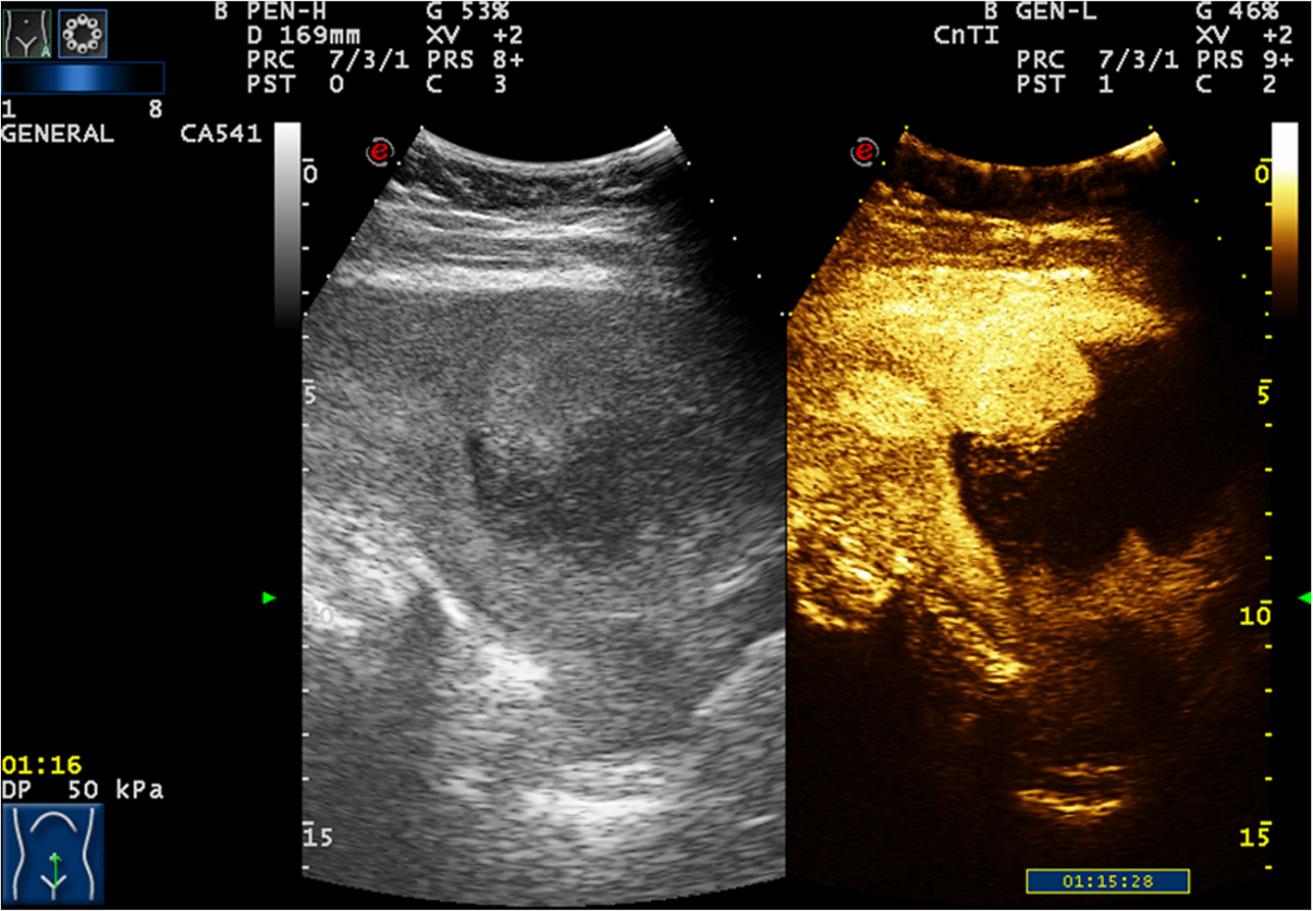
In the first case, the lesion demonstrated peripheral irregular hyper-enhancement in the arterial phase and later phases. The internal honeycomb-like structure partially showed hyper-enhancement. Remarkable non-enhancement was appeared in central area of lesion

A 32-year-old woman with amenorrhea for 2 months and vaginal bleeding for half a month was referred to our hospital. The first TVS performed in another hospital showed a heterogeneous mass on the anterior portion of the lower uterine segment measuring 67 * 66 mm, protruding into the uterine cavity. The blood flow signal was observed inside. The mass was considered as CSP. Blood tests performed on admission revealed a β-hCG value greater than 225,000 IU/ml (normal value, < 5.3 IU/ml). The ultrasound exam in our hospital showed an inhomogeneous hypoechoic lesion measuring 9.6 * 6.7 * 8.5 cm in the lower anterior part of the uterine. The boundary of the mass was indistinct with a honeycomb-like structure in the lesion and the mass compressed the uterine cavity obviously. the uterine cavity. Colour Doppler flow imaging (CDFI) showed abundant blood flow signals and a low resistive index (RI = 0.4) in the lesion. The lesion demonstrated peripheral irregular hyper-enhancement in the arterial phase. The honeycomb-like structure partially showed hyper-enhancement during all phases of the exam. Remarkable non-enhancement was appeared in central area of lesion (Fig. 1). CEUS suggested choriocarcinoma as the diagnosis, but incisional pregnancy combined with a partial hydatidiform mole was not excluded. MRI examination also suggested a trophoblastic tumour as the diagnosis. After prophylactic uterine artery embolization, hysteroscopy revealed that the anterior wall of the isthmus of the uterus showed prominent tumours, the mucosal surface was intact, and the uterine cavity was deformed by compression. No obvious neoplasms were observed in the uterine cavity. After 7 rounds of EMA and EP chemotherapy, laparoscopic total hysterectomy and bilateral salpingectomy was performed. Intraoperative anatomical specimens included 5 * 5 * 4 cm masses from the isthmus of the uterus. Major pathology showed (uterine) extensive necrosis with foam cell aggregation and inflammatory cell infiltration, consistent with changes after chemotherapy, chronic cervical mucosa inflammation, and (bilateral) fallopian tube tissue. Postoperative EMA and EP chemotherapy was continued. The latest blood tests revealed a β-hCG value of 1.1 IU/ml.

### Case two

A 34-year-old woman was referred to our hospital for amenorrhea without an obvious cause 3 months prior. Ultrasound examination in another hospital showed an uneven-echo mass measuring 4.1 * 3.5 cm from the lower uterus to the anterior wall of the cervix with an abundant blood supply. At that time, the β-hCG level was 72,587 U/ml (normal value, < 5.3 IU/ml). After considering intrauterine pregnancy, uterine artery embolization was performed. Then, ultrasound-guided curettage was performed. Postoperative pathology showed intrauterine placental tissue, and postoperative vaginal bleeding continued. Later, in our hospital, TVS showed that the mass near the lower anterior wall of the uterus was approximately 3.8 * 3.2 cm in size. CDFI revealed peripheral rich blood flow signals in the lesion. The β-hCG value was 184.3 mIU/ml (normal value, < 5.3 IU/ml). The initial diagnosis was suspicious of trophoblastic tumours with conventional ultrasound. However, analysis of the MRI data suggested a scar pregnancy. Laparoscopic pelvic uterine scar lesion resection and uterine repair was performed. During the operation, some omentum and the anterior peritoneum were found to have formed tight muscular adhesions, and the lower part of the anterior wall of the uterus near the cervix observed as a 4 * 3 cm bulge. Pathology showed necrotic tissue (uterine scar pregnancy tissue), fibres, inflammatory cells infiltration in the muscle tissue, and villi in a small amount of decidual tissue and necrotic tissue.

## Discussion and conclusions

Here, we have reported two cases of uterine lesions. Two patients were misdiagnosed after CS based on the first ultrasound exam. The first case of a trophoblastic tumour was initially diagnosed as a scar pregnancy, while the second case, which had a scar pregnancy, was misdiagnosed as a trophoblastic tumour. The misdiagnoses were due to the particularity of the location of the lesions in the two patients, namely, the low anterior uterine segment, complicating the ultrasound-based diagnosis and further hindering early clinical diagnosis and treatment. Therefore, understanding the characteristics of scar pregnancy and trophoblastic tumours is very important.

The clinical ultrasound diagnosis of uterine lesions is based mainly on the characteristics of the grayscale and colour Doppler ultrasound [16]. Recently, a clinical trial named TITANIUM aimed to describe the ultrasound features of GTN and to identify ultrasound predictors of resistance to single-drug chemotherapy in low-risk patients [17]. The typical imaging features of GTN lesions located at myometrium on ultrasound show heterogeneous echogenicity, which are sponge-like or honeycomb-shaped. CDFI showed increased vascularity within the masses which was caused by the presence of intralesional arteriovenous shunts. On spectral Doppler US, the vessels demonstrated a high-velocity, low-resistance waveform, mostly between 0.2 and 0.4. CDFI can aid in the evaluation of GTN since these lesions have arteriovenous communications [18]. In a prospective analysis of 246 women with complete mole, the Doppler pulsatility index showed potential as a predictor of subsequent development of GTN [19]. However, the US features of the atypical or special location of GTN may occasionally overlap with those of ectopic pregnancy [20].

Ultrasound is the first-line imaging modality diagnosing CSP. However, the sonographic imaging features of CSP are complex. Early diagnosis of CSP is not always achieved, and many cases are misdiagnosed as threatened or missed/incomplete miscarriage or simply intrauterine pregnancy. Such a misdiagnosis may lead to sharp curettage for a presumed failed pregnancy, which can result in profuse bleeding and emergency surgical intervention, on occasionally ending with hysterectomy. Residual villous tissue due to improper curettage continues to grow and infiltrate the myometrium, forming mass-based CSP. This kind of CSP is often depicted as a heterogeneous mass consisting of different components in the anterior wall of the lower uterine segment [21]. Under these conditions, CSP is difficult to differentiate from trophoblastic tumours [22]. In our second case, a highly vascularised heterogeneous mass was detected by TVS leading to a misdiagnosis of a trophoblastic tumour as in a previous study [23].

**Fig. 2:**
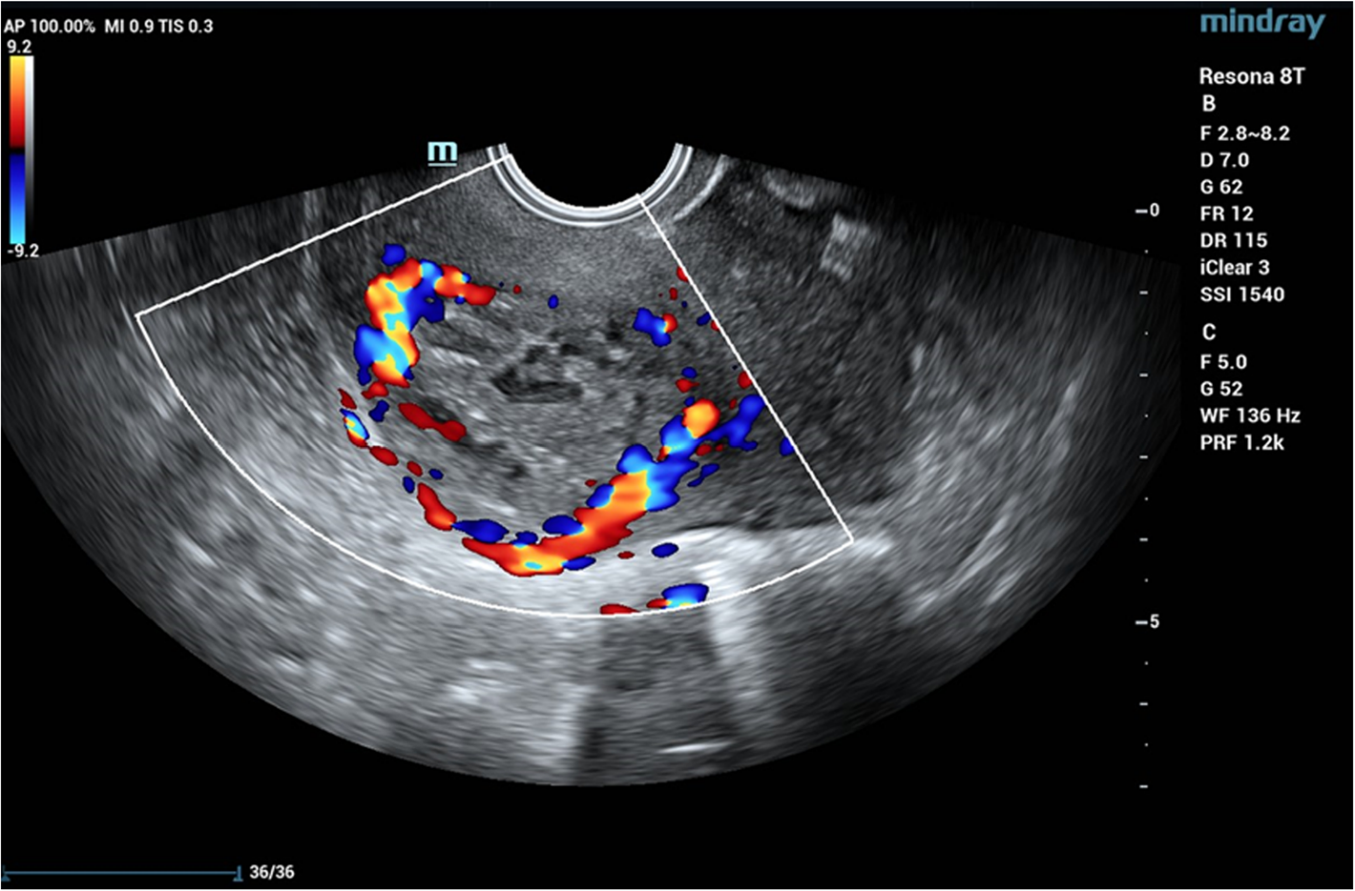
In the second case, the mass was located in the uterine isthmus. CDFI revealed peripheral rich blood flow signals in the lesion

CEUS is widely used in clinical practice and involves a microbubble contrast agent injected as a bolus through elbow veins to detect vascularity of the target lesion with obvious advantages in blood flow imaging [24]. Ultrasound images of CSP show a mass with peripheral rich blood flow in the cesarean section scar. After contrast injection, the lesions can be significantly hyper-enhanced. Regarding the features of a rich blood flow, CEUS has demonstrated superior diagnostic and classification efficacy for CSP compared to TVS due to more precise delineation of the gestational sac (GS) location, range, depth, and microcirculation pattern [25]. In the first case, conventional ultrasound was used to detect large masses in uterine isthmus, and CDFI showed rich blood flow signals around the masses. CEUS showed rapid hyper-enhancement of the area around the mass in the arterial phase, while the venous phase retreated slightly and still showed hyper-enhancement. Combined with the patient’s medical history and the location of the lesion, CEUS revealed a high probability of a trophoblastic tumour diagnosis. Unfortunately, the second patient was not examined by CEUS. Because the patient had a history of irregular vaginal bleeding and a uterine isthmus mass with a rich blood flow signal (Fig. 2), the lesion was misdiagnosed as a trophoblastic tumour after TVS.

GTD will produce excessive β-hCG, resulting in a level 3-100 times higher than that in a normal pregnancy, and the difference between an ectopic pregnancy and a normal intrauterine pregnancy is an abnormally slow increase or decrease in the β-hCG level. GTN should be considered when the patient has an inadequate decline in serum β-hCG level after curettage of a molar pregnancy or a high β-hCG level, especially more than 100 000 IU/L. The diagnosis of CSP only bases on a positive pregnancy test and the sonographic criteria [16]. Correlations with β-hCG levels are vital to performing a differential diagnosis that considers other conditions that can mimic a GTN, such as fibroids, retained products of conception, pelvic inflammatory disease, arteriovenous malformations and other uterine malignancies. Both patients later underwent MRI examinations, combined with β-hCG measurements, to establish the correct diagnoses. MRI can serve as a supplementary method to achieve the precise diagnosis at this time. If MRI still cannot confirm the diagnosis, hysteroscopy needs to be carefully performed, and preventive uterine artery embolization should be performed if necessary.

These two cases with completely different diagnoses had extremely similar ultrasound images and clinical presentations, thus complicating the clinical diagnosis. Therefore, in clinical practice, for women of childbearing age who present with irregular vaginal bleeding, abdominal pain, pelvic masses, and abnormal blood β-hCG levels, the possibility of CSP or GTN in special parts of the uterus must be considered when determining a differential diagnosis. In patients with irregular vaginal bleeding after a previous CS, when TVS shows a mass in the lower anterior wall of the uterus, the relationship between the mass and the uterine scar should be considered. When the mass is too large to be distinguished, further MRI can be considered to determine the specific location of the mass. If the lesion is located in the uterine scar and the blood β-hCG level is slightly elevated or decreased, a scar pregnancy can be considered, but if the lesion is not located in the uterine scar but in the myometrium and abnormal increases in blood β-hCG levels are evident, GTN should be considered. However, if MRI shows that the lesion is located in the uterine scar and the patient’s blood β-hCG is abnormally high, a rarer disease should be considered, such as GTN at the caesarean scar.

## Data Availability

Not applicable

## Abbreviations

GTN: Gestational trophoblastic neoplasia
WHO: World Health Organization
CS: Caesarean section
CSP: Caesarean scar pregnancy
CDFI: Colour Doppler Flow Imaging
RI: Resistance Index
CEUS: Contrast-enhanced ultrasonography
MRI: Magnetic resonance imaging
TVS: Transvaginal ultrasonography
GS: Gestational sac

## References

[1] Lumbiganon P, Laopaiboon M, Gulmezoglu AM, Souza JP, Taneepanichskul S, Ruyan P, Attygalle DE, Shrestha N, Mori R, Nguyen DH (2010). Method of delivery and pregnancy outcomes in Asia: the WHO global survey on maternal and perinatal health 2007-08. Lancet.

[2] Cali G, Timor-Tritsch IE, Palacios-Jaraquemada J, Monteaugudo A, Buca D, Forlani F, Familiari A, Scambia G, Acharya G, D’Antonio F (2018). Outcome of Cesarean scar pregnancy managed expectantly: systematic review and meta-analysis. Ultrasound Obstet Gynecol.

[3] Rajakumar C, Agarwal S, Khalil H, Fung Kee Fung KM, Shenassa H, Singh SS (2015). Caesarean scar pregnancy. J Obstet Gynaecol Can.

[4] Khunda A, Tay J (2007). Caesarean scar pregnancy. BJOG: an international journal of obstetrics gynaecology.

[5] Tantbirojn P, Crum C, Parast M (2008). Pathophysiology of placenta creta: the role of decidua and extravillous trophoblast. Placenta.

[6] Miller R, Timor-Tritsch I, Gyamfi-Bannerman C (2020). Society for Maternal-Fetal Medicine (SMFM) Consult Series #49: Cesarean scar pregnancy. American journal of obstetrics gynecology.

[7] Timor-Tritsch I, Monteagudo A, Santos R, Tsymbal T, Pineda G, Arslan A (2012). The diagnosis, treatment, and follow-up of cesarean scar pregnancy. American journal of obstetrics gynecology.

[8] Rotas MA, Haberman S, Levgur M (2006). Cesarean scar ectopic pregnancies: etiology, diagnosis, and management. Obstet Gynecol.

[9] Timor-Tritsch IE, Monteagudo A (2012). Unforeseen consequences of the increasing rate of cesarean deliveries: early placenta accreta and cesarean scar pregnancy. A review. Am J Obstet Gynecol.

[10] Birch Petersen K, Hoffmann E, Rifbjerg Larsen C, Svarre Nielsen H (2016). Cesarean scar pregnancy: a systematic review of treatment studies. Fertil Steril.

[11] Qian ZD, Zhu XM (2014). Caesarean scar choriocarcinoma: a case report and review of the literature. Eur J Med Res.

[12] Sorbi F, Sisti G, Pieralli A, Di Tommaso M, Livi L, Buccoliero A, Fambrini M (2013). Cervicoisthmic choriocarcinoma mimicking cesarean section scar ectopic pregnancy. Journal of research in medical sciences: the official journal of Isfahan University of Medical Sciences.

[13] Goldstein DP, Berkowitz RS (2012). Current management of gestational trophoblastic neoplasia. Hematol Oncol Clin North Am.

[14] Committee FO (2002). FIGO staging for gestational trophoblastic neoplasia 2000. FIGO Oncology Committee. Int J Gynaecol Obstet.

[15] Wang X, Li Y, Yang J, He Y, Wang M, Wan X, Xiang Y (2018). Identification and treatment of gestational trophoblastic neoplasia located in the cesarean scar. Int J Gynaecol Obstet.

[16] Timor-Tritsch IE, Monteagudo A, Cali G, D’Antonio F, Kaelin Agten A (2019). Cesarean Scar Pregnancy: Diagnosis and Pathogenesis. Obstet Gynecol Clin North Am.

[17] Verri D, Pasciuto T, Epstein E, Fruscio R, Mascilini F, Moro F, Scambia G, Valentin L, Testa AC (2019). GestaTIonal TrophoblAstic NeoplasIa Ultrasound assessMent: TITANIUM study. Int J Gynecol Cancer.

[18] Lin LH, Bernardes LS, Hase EA, Fushida K, Francisco RP (2015). Is Doppler ultrasound useful for evaluating gestational trophoblastic disease?. Clinics.

[19] Asmar FTC, Braga-Neto AR, de Rezende-Filho J, Villas-Boas JMS, Charry RC, Maesta I (2017). Uterine artery Doppler flow velocimetry parameters for predicting gestational trophoblastic neoplasia after complete hydatidiform mole, a prospective cohort study. Clinics.

[20] Dhanda S, Ramani S, Thakur M (2014). Gestational trophoblastic disease: a multimodality imaging approach with impact on diagnosis and management. Radiol Res Pract.

[21] Rygh AB, Greve OJ, Fjetland L, Berland JM, Eggebo TM (2009). Arteriovenous malformation as a consequence of a scar pregnancy. Acta Obstet Gynecol Scand.

[22] Imbar T, Bloom A, Ushakov F, Yagel S (2003). Uterine artery embolization to control hemorrhage after termination of pregnancy implanted in a cesarean delivery scar. J Ultrasound Med.

[23] Weimin W, Wenqing L (2002). Effect of early pregnancy on a previous lower segment cesarean section scar. Int J Gynaecol Obstet.

[24] Sidhu PS, Cantisani V, Dietrich CF, Gilja OH, Saftoiu A, Bartels E, Bertolotto M, Calliada F, Clevert DA, Cosgrove D (2018). The EFSUMB Guidelines and Recommendations for the Clinical Practice of Contrast-Enhanced Ultrasound (CEUS) in Non-Hepatic Applications: Update 2017 (Long Version). Ultraschall Med.

[25] Wu Y, Zhou L, Chen L, Zhou Q, Zeng T (2019). Efficacy of contrast-enhanced ultrasound for diagnosis of cesarean scar pregnancy type. Medicine.

